# Rescue Effects: Irradiated Cells Helped by Unirradiated Bystander Cells

**DOI:** 10.3390/ijms16022591

**Published:** 2015-01-23

**Authors:** R. K. K. Lam, Y. K. Fung, W. Han, K. N. Yu

**Affiliations:** 1 Department of Physics and Materials Science, City University of Hong Kong, Kowloon, Hong Kong; E-Mails: roykk.lam@my.cityu.edu.hk (R.K.K.L.); yikifung2-c@my.cityu.edu.hk (Y.K.F.); 2 Center of Medical Physics and Technology, Hefei Institutes of Physical Science, Chinese Academy of Sciences, Hefei 230031, China; E-Mail: hanw@hfcas.ac.cn; 3 State Key Laboratory in Marine Pollution, City University of Hong Kong, Kowloon, Hong Kong

**Keywords:** radioimmunotherapy, rescue effect, radiation induced bystander effect, intercellular signaling

## Abstract

The rescue effect describes the phenomenon where irradiated cells or organisms derive benefits from the feedback signals sent from the bystander unirradiated cells or organisms. An example of the benefit is the mitigation of radiation-induced DNA damages in the irradiated cells. The rescue effect can compromise the efficacy of radioimmunotherapy (RIT) (and actually all radiotherapy). In this paper, the discovery and subsequent confirmation studies on the rescue effect were reviewed. The mechanisms and the chemical messengers responsible for the rescue effect studied to date were summarized. The rescue effect between irradiated and bystander unirradiated zebrafish embryos *in vivo* sharing the same medium was also described. In the discussion section, the mechanism proposed for the rescue effect involving activation of the nuclear factor κB (NF-κB) pathway was scrutinized. This mechanism could explain the promotion of cellular survival and correct repair of DNA damage, dependence on cyclic adenosine monophosphate (cAMP) and modulation of intracellular reactive oxygen species (ROS) level in irradiated cells. Exploitation of the NF-κB pathway to improve the effectiveness of RIT was proposed. Finally, the possibility of using zebrafish embryos as the model to study the efficacy of RIT in treating solid tumors was also discussed.

## 1. Introduction

Conventional radioimmunotherapy (RIT) aims to deliver a lethal ionizing-radiation dose to tumor cells through a monoclonal antibody labeled with a radionuclide that has specificity for an antigen associated with the tumor cells. RIT has been an attractive tool for treating local and diffuse tumors with ionizing radiation. However, the efficacy of RIT might be compromised by a phenomenon called “rescue effect” which was discovered relatively recently by our group in 2011 [1].

Rescue effect is closely related to a more extensively studied non-targeted effect of ionizing radiation known as radiation-induced bystander effect (RIBE), which was first observed in *in vitro* experiments [2]. RIBE in cells referred to the phenomenon that unirradiated cells responded as if they had been irradiated after they had partnered with the irradiated cells or after they had been treated with the medium previously conditioning the irradiated cells. There are many reviews on RIBE (see reviews in e.g., refs. [3,4,5,6,7,8,9]). To date, two mechanisms underlying RIBE have been widely accepted, namely, (1) gap junction intercellular communication (GJIC) in the presence of physical contacts among the cells; and (2) communication of soluble signal factors among the cells through the shared medium. Various soluble signal factors that participate in RIBE have been proposed, including tumor necrosis factor-α (TNF-α) [10], transforming growth factor-β1 (TGF-β1) [11], interleukin-6 (IL-6) [12], interleukin-8 (IL-8) [13] and nitric oxide (NO) [14,15,16] and reactive oxygen species (ROS) [17].

The rescue effect describes the phenomenon where irradiated cells or irradiated organisms derive benefits from the feedback signals released from the bystander unirradiated cells or organisms. An example of the benefit is the mitigation of radiation-induced DNA damages. Chen *et al.* [1] discovered the rescue effect where the bystander cells, through sending intercellular feedback signals to the irradiated cells, mitigated the effects originally induced in the irradiated cells directly by the radiation. Chen *et al.* [1] found that the rescue effect reduced (1) the DNA double strand breaks (DSBs) surrogated by the numbers of p53-binding protein 1 (53BP1) foci; (2) the genomic instability surrogated by the number of micronucleus (MN) formation; and (3) the extent of apoptosis in the irradiated cells. In particular, the authors also revealed that unirradiated normal cells could rescue irradiated cancer cells.

**Figure 1:**
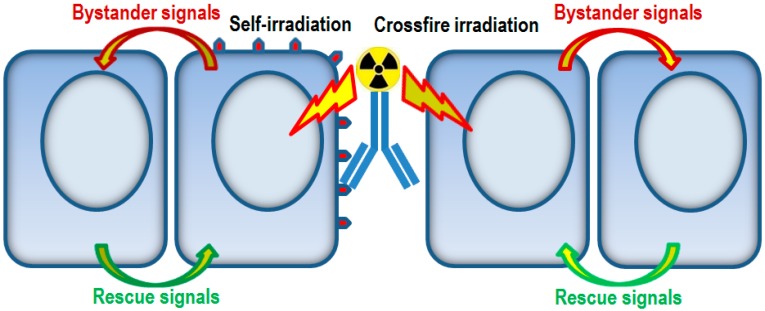
Schematic diagram showing the communication of bystander signals and rescue signals between unirradiated cells and irradiated cells, the latter having been irradiated by either self-irradiation (targeted cells) or crossfire irradiation (non-targeted cells) from the application of radioimmunotherapeutic agents.

As such, the efficacy of RIT can be compromised in the presence of rescue effect. Figure 1 is a schematic diagram showing the various effects on the cells involved after the application of radioimmunotherapeutic agents. In particular, targeted cells are irradiated by self-irradiation while non-targeted cells are irradiated by crossfire irradiation. Communication of bystander signals and rescue signals between unirradiated cells and irradiated cells will occur. While the rescue effect will compromise the efficacy of RIT, it will also help mitigate the damages in the non-targeted cells inflicted by crossfire irradiation.

In this paper, the discovery of rescue effect will first be reviewed in Section 2. Subsequent to this discovery, various other research groups succeeded in confirming the rescue effect in various cell systems. Widel *et al.* [18] observed the rescue effect in irradiated human melanoma (Me45) cells co-cultured with unirradiated normal human dermal fibroblasts (NHDF) cells. Pereira *et al.* [19] demonstrated the rescue effect between irradiated and unirradiated embryonic zebrafish fibroblast (ZF4) cells and Desai *et al.* [20] revealed the rescue effect in irradiated lung adenocarcinoma (A549) cells induced by partnered unirradiated human lung normal fibroblast (WI38) cells. These studies will be reviewed in Section 3.

Studies on the mechanisms and the chemical messengers responsible for communicating the rescue effect have been scarce. He *et al.* [21] confirmed the rescue effect between irradiated human macrophage U937 cells induced by bystander unirradiated HL-7702 hepatocyte cells, and demonstrated that the rescue effect was mediated by cyclic adenosine monophosphate (cAMP) through a membrane signaling pathway. Lam *et al.* [22] confirmed rescue effect between irradiated and unirradiated human cervical cancer HeLa cells, and proved the presence of a rescue signal in the medium having conditioned the bystander cells previously partnered with irradiated cells, which could activate the nuclear factor κB (NF-κB) response pathway in the irradiated cells. These mechanisms will be reviewed in Section 4.

The rescue effect was also discovered between irradiated and bystander unirradiated zebrafish embryos *in vivo* sharing the same medium [23]. Subsequently, Kong *et al.* [24] explored the properties of rescue signals, including the similarity in the functions of the bystander signals and the rescue signals, and the role played by NO and the NO-induced damages in the bystander effect and rescue effect between irradiated and unirradiated zebrafish embryos; these will be reviewed in Section 5. Finally, Section 6 will provide the overall discussion.

## 2. Discovery of Rescue Effect

In 2011, Chen *et al.* [1] discovered the rescue effect, which was closely related to RIBE. In fact, before this discovery, Goldberg and Lehnert had speculated that bystander cells could release their own signaling factors to affect the irradiated cells [5]. Chen *et al.* [1] observed the rescue effect in human primary fibroblast (NHLF) cells and cancer (HeLa) cells through the use of specially designed cell co-culture systems. The alpha-particle dose used was either 20 or 40 cGy (centi Gray). A number of biological endpoints were examined, including (1) the numbers of 53BP1 foci; (2) MN formation; (3) apoptosis levels and (4) survival, which are briefly reviewed below.

### 2.1. 53BP1 Foci in Irradiated Cells

Chen *et al.* [1] observed that α-particle irradiation of NHLF cells (IR_all_ cells) without co-culture with bystander cells or those (IR_by_ cells) co-cultured with bystander cells induced significant increases in the numbers of 53BP1-positive foci at 30 min post-irradiation, but with no statistically significant differences between the numbers of foci for IR_all_ and IR_by_ cells. By 24 h post-irradiation, the numbers of 53BP1 positive foci in IR_all_ or IR_by_ cells dropped significantly. In particular, the number of 53BP1 foci in IR_by_ cells was significantly smaller than that in IR_all_ cells, indicating that the bystander cells helped repair the DNA DSBs in the irradiated cells. The different manifestations of the rescue effect at 30 min and 24 h post-irradiation was likely due to the time required to facilitate the DNA repair. The irradiation conditions were designed to ensure that all cells in the designated irradiated population were actually irradiated, since any unirradiated cell would become a bystander cell. Contamination in the designated irradiated population with unirradiated bystander cells would lead to erroneous results, e.g., the rescue effect would then be present within the IR_all_ cell population itself.

### 2.2. MN Induction in Irradiated Cells

Chen *et al.* [1] revealed that α-particle irradiation of NHLF cells (without partnering with bystander cells) followed by 24-h incubation led to an increase in the ratio between the number of binucleated cells with MN to the total number of binucleated cells. In the presence of partnered bystander cells, however, the MN induction in the irradiated cells was significantly reduced, indicating that the bystander cells helped mitigate the MN induction in the irradiated cells.

### 2.3. Apoptosis and Survival in Irradiated Cells

The number of apoptotic cells, which were annexin V-positive (FL1-H), was significantly increased at 72 h post-irradiation. With partnered bystander cells, this number was significantly decreased [1]. As regards the colony formation assay, the surviving fraction *i.e.*, colonies counted/(cells seeded × plating efficiency), at 24 h post-irradiation of IR_all_ was lower than that of IR_by_, although the differences were not statistically significant. These results indicated that the bystander cells helped reduce the number of apoptotic irradiated cells and promoted survival of irradiated cells.

Chen *et al.* [1] also confirmed that unirradiated bystander NHLF cells significantly decreased the MN induction in the irradiated HeLa cells at 24 h post-irradiation, indicating that normal cells could also rescue irradiated cancer cells. This finding could have a far-reaching impact on the efficiency of treatment of cancers using ionizing radiation.

## 3. Other Studies Confirming the Rescue Effect 

Subsequent to our discovery of the rescue effect, various other groups confirmed the presence of the rescue effects in different cell systems.

### 3.1. Rescue Effect in Human Melanoma (Me45) Cells and Human Dermal Fibroblasts (NHDF)

Widel *et al.* [18] confirmed the rescue effect in irradiated human melanoma (Me45) cells co-cultured with non-irradiated normal human dermal fibroblasts (NHDF) cells using a transwell insert culture system, through the reduction in MN induction frequencies as well as apoptosis. However, the authors revealed that non-irradiated Me45 cells did not rescue co-cultured irradiated Me45 cells or co-cultured irradiated NHDF cells. The authors also did not detect significant (despite finding an indication of) rescue effect in irradiated fibroblasts by non-irradiated fibroblasts. This was in contrast to the results of Chen *et al.* [1] who observed the rescue effect between human primary fibroblast (NHLF) cells, and the results of Pereira *et al.* [19] who observed the rescue effect between irradiated and unirradiated embryonic zebrafish fibroblast (ZF4) cells. The discrepancies were likely due to the different doses employed. While Widel *et al.* [18] used 2 or 4 Gy of 6 MV X-rays for irradiation, Chen *et al.* [1] used 20 or 40 cGy of alpha-particle doses, and Pereira *et al.* [19] used 70 or 550 mGy (milli Gray) of gamma-ray doses from a ^137^Cs gamma irradiator.

Furthermore, Widel *et al.* [18] found that the rescue effect in the irradiated Me45 cells co-cultured with non-irradiated NHDF cells tied in with a substantial decrease in the ROS level in the irradiated cells, when compared to the ROS levels in irradiated Me45 cells co-cultured with non-irradiated Me45 cells or those without co-cultured bystander cells. The ROS level in irradiated NHDF cells co-cultured with non-irradiated NHDF cells was also reduced. The mechanism underlying the changes in the ROS level was not studied by the authors.

### 3.2. Rescue Effect in Embryonic Zebrafish Fibroblast (ZF4) Cells

**Figure 2:**
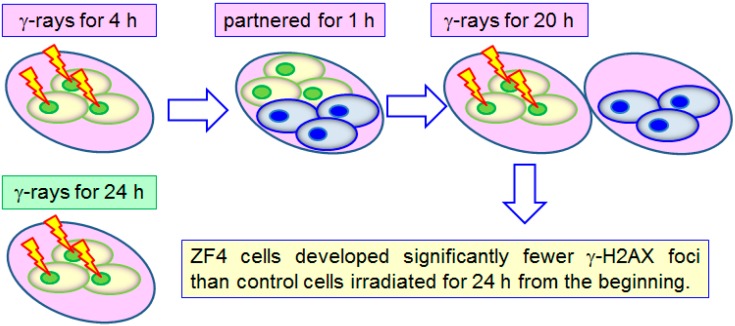
Comparison of γ-H2AX foci developed on embryonic zebrafish (ZF4) cells upon two different treatments: Top row: gamma radiation for 4 h, then partnered them with non-irradiated bystander ZF4 cells for 1 h, and finally irradiated with gamma radiation for another 20 h; bottom row: gamma radiation for 24 h. Treatment in the top row led to significantly fewer γ-H2AX foci than treatment in the bottom row.

Pereira *et al.* [19] compared the number of γ-H2AX foci that developed in embryonic zebrafish fibroblast (ZF4) cells with two different treatments: (a) gamma radiation for 4 h with a total dose of 12 mGy (dose rate of 70 mGy/day) or 92 mGy (dose rate of 550 mGy/day) using a ^137^Cs gamma irradiator, then partnered them with non-irradiated bystander ZF4 cells for 1 h, and finally irradiated them with gamma radiation for another 20 h with a total dose of 58 mGy (dose rate of 70 mGy/day) or 460 mGy (dose rate of 550 mGy/day); (b) gamma radiation for 24 h. Schematic diagrams showing the two different treatments are shown in Figure 2. The authors found that treatment (a) led to significantly fewer γ-H2AX foci than treatment (b), and as such, the rescue effect between ZF4 cells was also successfully demonstrated. The 1-h gap might have helped the cells develop some kind of adaptation, so the significantly fewer γ-H2AX foci for treatment (a) could have been a combined effect of rescue effect and radioadaptation.

### 3.3. Rescue Effect in Lung Adenocarcinoma (A549) Cells and Human Lung Normal Fibroblast (WI38) Cells

**Figure 3:**
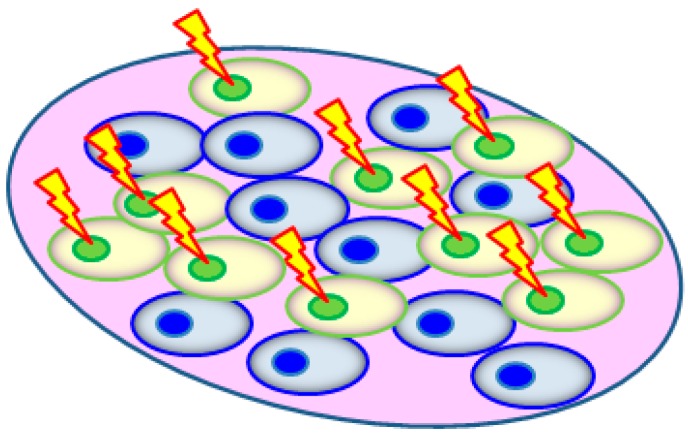
The cells (A549 or WI38) to be irradiated by microbeam protons were labeled with cell tracker orange (CTO) 1 day before irradiation, which were then mixed with unlabeled A549/WI38 cells in a 1:1 ratio.

Desai *et al.* [20] adopted the cell co-culture approach to study the bystander effects and rescue effects between lung adenocarcinoma (A549) cells and human lung normal fibroblast (WI38) cells. The desired radiation dose was delivered using microbeam protons (energy = 3.4 MeV; LET = 11.7 keV/µm; beam diameter ~2 µm) at room temperature using the Single Particle Irradiation System to Cells (SPICE) facility at the National Institute of Radiological Sciences (NIRS), Chiba, Japan. One day before the experiments, the A549 or WI38 cells to be irradiated were first labeled with cell tracker orange (CTO) and then mixed with unlabeled A549/WI38 cells in a 1:1 ratio. Each nucleus of the CTO-labelled cells was then irradiated with 500 microbeam protons that were delivered within 0.05 s (Figure 3).

The number of γ-H2AX foci fluorescence intensity per cell nucleus was chosen as the studied biological endpoint. The authors showed that the γ-H2AX foci fluorescence intensity per nucleus of irradiated A549 cells partnered with unirradiated A549 cells surged significantly with time, with the peak value recorded at 3 h post-irradiation significantly higher than the corresponding value obtained from a co-culture with bystander WI38 cells. These latter observations suggested mitigation of the proton-induced DNA damage in the irradiated A549 cells by the bystander WI38 cells, which hinted at a rescue effect provided by these WI38 cells, or that bystander WI38 cells provided a much stronger rescue effect than bystander A549 cells. 

Despite the occurrence of the rescue effect, the authors did not observe detrimental bystander effects on the bystander WI38 cells induced by the irradiated A549 cells. This likely showed that the bystander signals from the irradiated cells, which triggered the generation of rescue signals in the bystander cells, might not necessarily lead to observable damages. Relatedly, Kong *et al.* [24] also found that unirradiated zebrafish embryos needed NO, which was a common soluble molecule found to play an important role in the communication of the bystander effect, but not the NO-induced damage, in order to rescue their partnered α-particle irradiated zebrafish embryos.

The authors also studied the involvement of GJIC in the rescue effect from bystander WI38 cells to irradiated A549 cells, knowing that GJIC existed between WI38 and A549 cells. Before irradiation, the A549 cells were treated with lindane to block the GJIC. However, this lindane treatment did not significantly alter the γ-H2AX foci intensity per irradiated A549 nucleus, which suggested that GJIC was not involved in this rescue effect, and that probably soluble factors might play an important role.

On the other hand, the authors did not observe significant changes in the γ-H2AX foci intensity per irradiated WI38 nucleus regardless of partnering with WI38 or A549 cells, which meant no rescue effect was induced by bystander A549 cells on irradiated WI38 cells.

## 4. Mechanisms Underlying the Rescue Effect

### 4.1. Involvement of cAMP

He *et al.* [21] examined a possible mechanism underlying the rescue effect, which involved the second messenger cyclic adenosine monophosphate (cAMP). The authors confirmed the presence of rescue effect between alpha-particle-irradiated human macrophage U937 cells (with an inactive p53) and bystander unirradiated HL-7702 hepatocyte cells (with wild-type p53) co-cultured with one another for 6 h by showing that (1) the mitochondria depolarization associated with apoptosis was induced in the bystander cells; and (2) the frequencies of MN formation in the irradiated cells were decreased. Alpha particles were delivered using an ^241^Am source with a dose of 40 cGy at a dose rate of 0.244 Gy/min. The energy of the alpha particles reaching the cells was 4.4 MeV which corresponded to a Linear Energy Transfer (LET) of 100 keV/µm.

**Figure 4:**
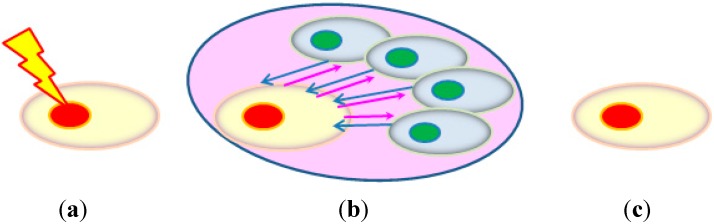
The co-culture method used by He *et al.* [21] to study the rescue effect. Before co-culturing, the irradiated and bystander cells were stained with the red fluorescent dye DiIC_18_ and the green fluorescent dye DiOC_18_, respectively. (**a**) Cell irradiation before co-culturing; (**b**) co-culturing to allow communication of bystander signals (pink arrows) and rescue signals (blue arrows) between the irradiated cell (with red nucleus) and the bystander cells (with green nuclei); and (**c**) irradiated cells washed away from the cell co-culture for examination after the desired co-culture period.

He *et al.* [21] used the cell co-culture method to allow the communication of bystander signals and rescue signals between the irradiated and bystander cell, the schematic diagrams of which are shown in Figure 4. Before co-culturing the irradiated and bystander cells, they were stained with the red fluorescent dye DiIC_18_ and the green fluorescent dye DiOC_18_, respectively. After the desired co-culture period, the red fluorescent irradiated cells were completely washed away from the cell co-culture with PBS for examination. Without the co-culture, the cAMP level in the irradiated cells rapidly increased at the beginning and peaked at 30 min after irradiation, then gradually decreased to the lowest level at 6 h after irradiation, and stabilized until 12 h. After the 6-h co-culture, the intracellular cAMP level in the irradiated cells was recovered while that in the bystander cells was reduced. He *et al.* [21] proposed that the decrease in the cAMP level in the irradiated cells as a result of irradiation was compensated by the cAMP from the bystander cells.

Furthermore, He *et al.* [21] demonstrated that cAMP communicated from the bystander cells to the irradiated cells mitigated the radiation damages in the latter through the treatment of bystander cells with forskolin (an activator of adenylyl cyclase, and cAMP was synthesized from adenosine triphosphate (ATP) by adenylyl cyclase) or KH-7 (an inhibitor of adenylyl cyclase) 2 h before the co-culture, and also showed that cAMP deficiency in the bystander cells enhanced their apoptosis. The function of cAMP was also confirmed through achieving a similar rescue effect by treating the irradiated cells with exogenous cAMP for 6 h after irradiation. Judging from these results, He *et al.* [21] proposed that the rescue effect was mediated by cAMP communicated from bystander cells to irradiated cells. Interestingly, even when the bystander cells had insufficient cAMP, they would still supply cAMP to the irradiated cells, and the resulting cAMP level in the irradiated cells would still be slightly higher than the corresponding value in the absence of co-cultured bystander cells. With this cell co-culture setup, it would be convenient to study the participation of various pathways in the bystander cells in the rescue effect by treating the bystander cells before the co-culture. However, it would be difficult to study the participation of various pathways in the irradiated cells in the rescue effect upon receiving the rescue signal, since any treatment might lead to the potential ambiguity of whether the effects were on rescue signals or on bystander signals (see Figure 4b).

He *et al.* [21] further suggested that cAMP was communicated through a membrane signaling pathway, and from the bystander cells to the irradiated cells, by showing that the rescue effect was abrogated when the cell membrane signaling pathway was blocked by filipin. Judging from all these observations, the authors concluded that bystander cells released cAMP to refill the irradiated cells with cAMP through a membrane signaling pathway and that this cAMP communication was crucial for the rescue effects.

Although cAMP could help protect cells against radiation-induced DNA damages [25], the underlying mechanisms were not fully understood. Naderi *et al.* [26] proposed that elevated cAMP levels enhanced the binding of p53 to its negative regulator HDM2, which overrode the stabilization of the p53 protein induced by DNA damages. It was established that p53 induction could activate the apoptotic program. However, the causal relationship between the increase in cAMP levels and the enhancement in p53 binding to HDM2 was not fully established. Involvement of other intermediate steps was not ruled out. One example would be the involvement of the NF-κB pathway. As described in more details in Section 6.1 below, cAMP can increase the affinity of RelA (p65) (a member of the NF-κB family) for transcriptional co-activators, while the IκB-kinase subunit beta (IKK2)/RelA could up-regulate the Mouse Double Minute 2 homolog (MDM2) expression to antagonize the p53 pathway [27].

### 4.2. NF-κB Activation

Lam *et al.* [22] used the “conditioned medium” approach to confirm the presence of rescue effect and to study the role of NF-κB activation in the irradiated cells induced by the bystander cells in the rescue effect. Here, the irradiated cells receiving the rescue signals were not the same as the irradiated cells releasing the bystander signals, and the rescue signals were present in the medium (hereafter referred to as CM) having conditioned the bystander cells previously partnered with irradiated cells releasing the bystander signals. As such, the irradiated cells receiving the rescue signals were not partnered with other cells. The NF-κB activation inhibitor BAY-11-7082, which blocked TNF-α-induced phosphorylation of IκBα, was employed to prove the participation of NF-κB in the rescue effect.

The authors used the human cervical cancer HeLa cells for their studies, employed alpha particles for irradiation and relied on the number of 53BP1 foci/cell as the studied biological endpoint. The alpha-particle dose of 5 cGy was delivered using an ^241^Am source (average alpha particle energy = 5.16 MeV, activity = 5.02 µCi, dose rate = 18 cGy/min). In fact, alpha-particle irradiation was utilized for two separate purposes, namely, (1) to prepare irradiated cells on which the effects of different treatments would be studied; or (2) to prepare the CM. Alpha-particle irradiation of cells always took place in a “Mylar-film dish”, which was a 100 mm diameter tissue culture dish with a hole of 10 mm diameter at the center and covered by a Mylar film with a thickness of 3.5 µm. The thin Mylar film allowed the alpha particles to go through without causing significant energy losses in the alpha particles.

**Figure 5:**
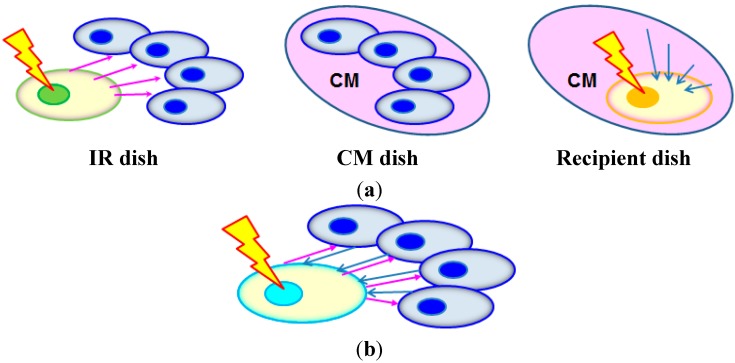
(**a**) Special procedures to study the rescue effect by physically separating the rescue signals (represented by blue arrows) from the bystander signals (represented by pink arrows) through the preparation of the CM (pink). The irradiated cell (green) sending out the bystander signals was different from the irradiated cell (orange) receiving the rescue signals. Preparation and use of the CM involved three separate dishes. The first was the “IR dish” to hold the cells for alpha-particle irradiation with partnered unirradiated cells to allow co-culture. The second was the “CM dish” to hold the unirradiated cells transferred from the IR dish to create the CM. The third was the “Recipient dish” to hold the HeLa cells for alpha-particle irradiation, which were immersed in the CM harvested from the CM dish; (**b**) Setup to study the rescue effect without physically separating the rescue signals from the bystander signals. The irradiated cell (cyan) receiving the rescue signals was the same as the irradiated cell sending out the bystander signals.

Preparation of the CM was a special methodology designed to physically separate the rescue signals from bystander signals. This methodology is schematically shown in Figure 5a, together with a methodology which does not involve the preparation of CM as shown in Figure 5b. Briefly, preparation and use of the CM involved three separate dishes. The first was a “Mylar-film dish” to hold the HeLa cells for alpha-particle irradiation (5 cGy) with partnered unirradiated Hela cells to allow co-culture for 2 h, and this dish was referred to as the “IR dish”. To facilitate the transfer of the unirradiated Hela cells, these cells were plated on cover glasses placed on the bottom of the IR dish, instead of being directly plated on the bottom of the IR dish. The second was a tissue culture dish (without a hole at the center) with 15 mL fresh medium to hold the cover glasses plated with the unirradiated Hela cells transferred from the IR dish for another 2 h to create the CM, and this dish was referred to as the “CM dish”. The third was another “Mylar-film dish” known as the “Recipient dish” to hold the HeLa cells for alpha-particle irradiation (5 cGy), which were immersed in the CM harvested from the CM dish. By using the methodology in Figure 5a, it would be possible to study the effect on the rescue signals alone, viz., by adding an NF-κB activation inhibitor into the CM without the presence of the irradiated cells which sent out the bystander signals. This methodology would remove any potential ambiguity of whether the effects were on the rescue signals or on the bystander signals, as encountered in the methodology shown in Figure 5b.

**Figure 6:**
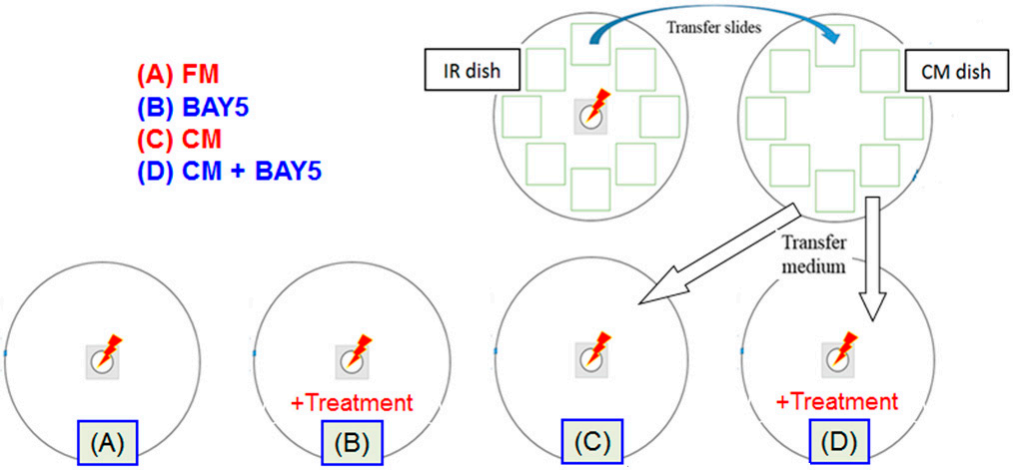
Experimental setup and procedures for the “conditioned medium” approach to prove the presence of rescue effect and to study the role of NF-κB activation in the irradiated cells by the bystander cells in the rescue effect. A total of six dishes were involved, including four “Mylar-film dishes” (**A** to **D**, lower row of dishes), the IR dish and the CM dish (upper row of dishes).

The experimental setup and procedures to prove the presence of rescue effect and to study the role of NF-κB activation in the rescue effect are shown in Figure 6. HeLa cells in the four “Mylar-film dishes” (A to D, lower row in Figure 6) were first irradiated with 5 cGy of α-particles and then subjected to four different treatments, including (A) treatment with 15 mL fresh medium (FM); (B) treatment with 15 mL FM + 5 µM BAY-11-7082 (referred to as BAY5); (C) treatment with 15 mL of the CM; and (D) treatment with 15 mL of (CM + BAY5). At 30 min later, the medium in the Mylar-film dishes (A) to (D) was replaced by FM and 53BP1 immuno-fluorescent staining was performed at 12 h post-irradiation.

Using the setup shown in Figure 6, Lam *et al.* [22] found significantly fewer 53BP1 foci/nucleus after treatment (C) when compared to the treatment (A), which confirmed the rescue effect in HeLa cells induced by alpha-particle irradiation (through the CM). The authors also noticed no significant differences upon treatments (A) and (B), so BAY-11-7082 did not affect the activation of NF-κB response pathway in the irradiated cells induced by direct irradiation alone. Finally, the authors revealed significantly more 53BP1 foci/nucleus after treatment (D) when compared to the treatment (C), which proved the presence of a rescue signal in the CM that could activate the NF-κB response pathway in the irradiated cells.

## 5. Rescue Effect between Irradiated and Bystander Zebrafish Embryos

Rescue effect was also induced between irradiated and bystander unirradiated zebrafish embryos (*Danio rerio*) [23,24]. All these studies involved irradiation of the zebrafish embryos with an α-particle dose of ~4.4 mGy from an ^241^Am source (with an α-particle energy of 5.49 MeV under vacuum and an activity of 4.26 kBq), and used the number of apoptotic signals on the irradiated embryos at 24 h post fertilization (hpf) as the studied biological endpoint. These results were particularly relevant to studies on human disease as the human and zebrafish genomes shared considerable homology, including conservation of most DNA repair-related genes [28]. In relation, zebrafish embryos were also a well-established model for studying DNA damages due to ionizing radiation [29,30,31,32,33,34,35,36,37,38].

Choi *et al.* [23] employed the “partnering” approach and discovered the rescue effect between irradiated and bystander unirradiated zebrafish embryos sharing the same medium, in terms of a significant reduction in the number of apoptotic signals on the irradiated embryos. The authors also found that the strength of the rescue effect significantly increased with the number of rescuing bystander unirradiated embryos.

Kong *et al.* [24] further explored the properties of the rescue signals involved through the “medium transfer” approach. The authors prepared an irradiated embryo conditioned medium (IECM) previously conditioned by 20 embryos irradiated at 5 hpf, into which 10 irradiated zebrafish embryos were immersed. The apoptotic signals in the 10 irradiated zebrafish embryos treated with the IECM were significantly fewer than those treated with the sham-irradiated embryo conditioned medium (SECM) provided by another 20 sham-irradiated zebrafish embryos. Besides showing the involvement of a released signal in rescue effect, the results also revealed that the signals released by the irradiated embryos, *i.e.*, the bystander signals by definition, were performing functions similar to the rescue effect on other irradiated embryos. The latter result strongly suggested that similarity between the bystander and rescue signals, but probably with different concentrations.

Furthermore, Kong *et al.* [23] studied the role played by NO through use of the NO scavenger cPTIO (2-(4carboxyphenyl)-4,4,5,5-tetramethyl-imidazoline-1-oxyl-3-oxide), as well as that played by NO-induced damages through the use of carbon monoxide (CO) released from CORM-3 (tricarbonylchloro(glycinato)ruthenium (II)) in radiation induced bystander effect and rescue effect between irradiated and unirradiated zebrafish embryos. The authors found that the cPTIO treatment on zebrafish embryos completely suppressed both the bystander and rescue effects. In contrast, while CORM-3 treatment on zebrafish embryos completely suppressed the bystander effect, which agreed with the results of Choi *et al.* [39], it did not suppress the rescue effect. It was previously established that exogenous CO was able to protect the bystander cells against the toxicity of NO, which could react with superoxide anions to form peroxynitrite (ONOO^−^) to cause DNA damage and lipid oxidation [40], and thus lead to apoptosis. In conclusion, the unirradiated zebrafish embryos needed NO, which was essential for inducing bystander effects in the unirradiated embryos, to initiate the rescue effect, but did not need the NO-induced damages to initiate the rescue effect. Incidentally, Desai *et al.* [20] also reported the occurrence of a rescue effect induced in lung adenocarcinoma (A549) cells by human lung normal fibroblast (WI38) cells, without observable detrimental bystander effects on the bystander WI38 cells induced by the irradiated A549 cells.

## 6. Discussion 

### 6.1. Rescue Effects Observed in in Vitro Experiments and Underlying Mechanisms

To date, *in vitro* rescue effects have been confirmed between different combinations of irradiated cells and bystander cells, including human primary fibroblast (NHLF) cells and cancer (HeLa) cells [1], human melanoma (Me45) cells and normal human dermal fibroblasts (NHDF) cells [18], irradiated and unirradiated embryonic zebrafish fibroblast (ZF4) cells [19], lung adenocarcinoma (A549) cells and human lung normal fibroblast (WI38) cells [20], human macrophage U937 cells and HL-7702 hepatocyte cells [21], and irradiated and unirradiated human cervical cancel HeLa cells [22]. The biological endpoints studied included (1) the numbers of 53BP1 foci [1,22]; (2) γ-H2AX foci number or fluorescence intensity per cell nucleus [19,20]; (3) MN formation [1,18,21]; (4) apoptosis levels [1,18]; (5) survival [1] and (6) mitochondria depolarization [21].

The magnitude of the rescue effect (in terms of percentage of reduction in the radiation effects in the irradiated cells) varied significantly according to the cell types (irradiated and bystander cells), the biological endpoints and the radiation dose. For using the numbers of 53BP1 foci as the biological endpoint, the magnitude of the rescue effect was about 13% [1] and 8% [22]. For using γ-H2AX foci number or fluorescence intensity per cell nucleus as the biological endpoint, the magnitude was 62% to 89% [19]. For using MN formation as the biological endpoint, Chen *et al.* [1] found the magnitude to be 33% (for 20 cGy irradiation) and 25% (for 40 cGy irradiation); while Widel *et al.* [18] found the magnitudes for 2 Gy irradiation to be 13% and 46% (24 h post irradiation, for partnering irradiated Me45 cells with non-irradiated Me45 and NHDF cells, respectively), 7% and 50% (48 h post irradiation), and 7% and 63% (72 h post irradiation); and for 4 Gy irradiation to be 12% and 57% (24 h post irradiation), 4% and 47% (48 h post irradiation), 10% and 54% (72 h post irradiation). These results also highlighted the important influence of irradiation dose on the rescue effect. To date, the radiation dose response of the irradiated cells for the rescue effect has not been studied in detail, and such studies will be pertinent for understanding and for application of the rescue effect. Moreover, induction of rescue effect was not always significant. For examples, Chen *et al.* [1] found that the rescue of irradiated HeLa cells by bystander NHLF cells was not significant in terms of the surviving fraction in the HeLa cells. Widel *et al.* [18] revealed that non-irradiated Me45 cells did not significantly rescue co-cultured irradiated Me45 cells or co-cultured irradiated NHDF cells. Desai *et al.* [20] did not observe significant changes in the γ-H2AX foci intensity per irradiated WI38 nucleus regardless of partnering with WI38 or A549 cells.

As mentioned in Section 1, studies on the mechanisms and the chemical messengers responsible for communicating the rescue effect have been scarce. First the mechanisms should be able to explain the observed biological endpoints, including the promotion of repair of DNA damages (as reflected by the reduction in the numbers of 53BP1 foci and γ-H2AX foci, reduction in the γ-H2AX foci fluorescence intensity per cell nucleus, reduction in mitochondria depolarization, reduction in apoptosis and increase in survival), as well as the promotion of correct repair of DNA damages (as reflected by the reduction in the MN formation). The MN assay is commonly employed to characterize genomic damages, and has been a popular tool for illustrating the presence of rescue effect [1,18,21]. However, attempts to propose mechanisms for the rescue effect which explain the mitigation of genomic damages have been relatively scarce.

Moreover, Desai *et al.* [20] reported that the rescue effect between lung adenocarcinoma (A549) cells and human lung normal fibroblast (WI38) cells did not involve GJIC, despite that GJIC existed between WI38 and A549 cells. If this finding is generic, it is likely that the rescue effect is mediated by a soluble factor. Both the cAMP proposed by He *et al.* [21], as well as TNF-α (that induced NF-κB activation which was proposed by Lam *et al.* [22] to be involved in rescue effect) were soluble factors. Moreover, Widel *et al.* [18] found that the rescue effect in the irradiated Me45 cells co-cultured with non-irradiated NHDF cells tied in with a substantial decrease in the ROS level in the irradiated cells. To be compatible with all these previous findings, it would be desirable if the proposed mechanism is: (1) promoting cellular survival; (2) promoting correct repair of DNA damages (e.g., promoting homologous recombination (HR) over non-homologous end joining (NHEJ)); (3) cAMP-dependent; (4) able to modulate the intracellular ROS level in the irradiated cells. In the following, we would assess the compatibility with these findings of the mechanism involving activation of the NF-κB pathway proposed by Lam *et al.* [22] for the rescue effect.

(1) Expression of NF-κB target genes in general promotes cellular survival. The anti-apoptotic proteins regulated by NF-κB were reviewed by Magné *et al.* [41]. Moreover, as described in Section 4.1, IKK2/RelA could up-regulate the MDM2 expression to antagonize the p53 pathway [27], where p53 induction could activate the apoptotic program; (2) NF-κB plays important roles in DNA repair, particularly through HR. Multiple mechanisms for NF-κB-mediated HR were proposed [42]. In addition, the NF-κB-mediated HR balanced the repression of HR by p53 [43]; (3) cAMP can bind to and activate the cAMP-dependent protein kinase (or protein kinase A, PKA), which is normally inactive, to enable it to phosphorylate serine 276 on RelA (p65) (a member of the NF-κB family) once in the nucleus to increase its affinity for the transcriptional co-activators CBP/p300 [44], where CBP is the CREB binding protein and CREB is the cAMP response element binding protein. Moreover, as described in Section 4.1 and item (1) above, IKK2/RelA could up-regulate MDM2 expression to antagonize the p53 pathway [27]; (4) Certain NF-κB-regulated genes play important roles in regulating the intracellular ROS levels [45]. As such, the activation of the NF-κB pathway proposed by Lam *et al.* [22] for the rescue effect seemed to be compatible with all the previous findings.

### 6.2. Rescue Effects Observed between Zebrafish Embryos

As described in Section 5, Choi *et al.* [23] and Kong *et al.* [24] confirmed the induction of rescue effect between irradiated and bystander unirradiated zebrafish embryos. Moreover, Kong *et al.* [24] concluded that the bystander signals and rescue signals between the zebrafish embryos had similar functions. It would be natural to ask why the rescue would be more effective if the irradiated embryos were (a) partnered with unirradiated embryos (instead of other irradiated embryos); or (b) placed in an IECM which had conditioned more irradiated embryos. In general, activations of p53 and NF-κB are associated with promotion of and protection from apoptosis, respectively. Some examples have been summarized in Section 6.1 above. The anti-apoptotic proteins regulated by NF-κB were reviewed by Magné *et al.* [41]. The IKK2/RelA could up-regulate the MDM2 expression to antagonize the p53 pathway [27]. Moreover, p53 and NF-κB inhibited each other’s ability to stimulate gene expression, and that this process was controlled by the relative levels of each transcription factor [46]. As such, a copious amount of rescue signals could be strong enough to activate the NF-κB pathway and to override the p53 pathway, thereby promoting survival of the irradiated cells in the irradiated embryos. This conjecture also explained the observation that the rescue effect became stronger with a larger ratio between the number of rescuing bystander unirradiated embryos and the number of irradiated embryos [23].

### 6.3. Rescue Effect in Radioimmunotherapy

At the time of discovery of the rescue effect, it was already recognized that the effect would have far reaching consequences on the treatment procedures of tumors using ionizing radiation, particularly when it was discovered that unirradiated normal cells could rescue irradiated cancer cells [1]. As explained in Section 1, while the rescue effect will compromise the efficacy of RIT, it will also help mitigate the damages in the non-targeted cells inflicted by crossfire irradiation. From the above discussion, it is now understood that activation of the NF-κB pathway in irradiated cells is the crucial step for the rescue effect. In fact, NF-κB-dependent mechanisms were previously found to lead to resistance against genotoxic treatment in anti-cancer therapies [42,47,48]. In relation, immuno-deficiency patients with mutations in the NF-κB pathway were predicted to be more sensitive to genotoxic therapies [49]. Taken together, it appeared that considerable benefits would be derived from finding ways to exploit the activation or inactivation of the NF-κB pathway to improve the effectiveness of RIT in the future.

On the other hand, the rescue effect studied using zebrafish embryos *in vivo* might help illustrate the rescue effect induced by RIT within solid tumors or the rescue effect between tumors arising from micrometastatic disease targeted by RIT. Application of RIT to treat solid tumors has been an attractive idea because it can target both known and occult lesions. Although RIT has been successfully applied to treat lymphoma, it has been generally facing a number of obstacles in treating solid tumors including, among others, heterogeneities in blood flow, tumor stroma, expression of target antigens and radioresistance [50]. Indeed, very few trials to treat solid tumors by RIT have progressed beyond Phase II (see e.g., ref. [51]). Efforts have been continually devoted to developing and optimizing strategies to surmount the obstacles for and to enhance the clinical response of solid tumors to RIT (see e.g., refs. [50,51,52]). The zebrafish embryo model can be useful for studying the properties of rescue effect in these tumors including, e.g., the effects of non-uniform radiation doses delivered to the tumors. There is also a new trend to target antigens associated with signaling pathways which are critical for growth and survival of the tumor [52]. The zebrafish embryo model could also be helpful for studying radiation effects on tumors [38]. In fact, tumorigenesis and embryonic development are related to each other [53,54,55,56,57]. In the past few decades, the embryonic origin of tumors was established (e.g., see review in ref. [58]), and attempts to fight cancer from the perspective of developmental biology have been made [59,60].

## 7. Conclusions 

The present paper reviewed the discovery in 2011 and research progress of a phenomenon called the rescue effect where irradiated cells or irradiated organisms derived benefits from the feedback signals released from the bystander unirradiated cells or organisms. The rescue effect can compromise the efficacy of all radiotherapy including RIT, noting in particular that unirradiated normal cells can rescue irradiated cancer cells. The mechanisms and the chemical messengers involved in the rescue effect proposed to date were described. In particular, activation of the NF-κB pathway in irradiated cells has been identified as the crucial step for the rescue effect. The activation of the NF-κB pathway can also explain the promotion of cellular survival and correct repair of DNA damages, the dependence on cAMP and the modulation of intracellular ROS level in the irradiated cells, which have been observed in previous studies on the rescue effect. The rescue effect has also been observed between irradiated and bystander unirradiated zebrafish embryos, which may help illustrate the rescue effect induced by RIT within solid tumors or between tumors.
